# Plant Secondary Metabolites with an Overview of *Populus*

**DOI:** 10.3390/ijms22136890

**Published:** 2021-06-26

**Authors:** Ali Movahedi, Amir Almasi Zadeh Yaghuti, Hui Wei, Paul Rutland, Weibo Sun, Mohaddeseh Mousavi, Dawei Li, Qiang Zhuge

**Affiliations:** 1 Co-Innovation Center for Sustainable Forestry in Southern China, Key Laboratory of Forest Genetics & Biotechnology, Ministry of Education, College of Biology and the Environment, Nanjing Forestry University, Nanjing 210037, China; amir_20364@yahoo.com (A.A.Z.Y.); 15850682752@163.com (H.W.); czswb@njfu.edu.cn (W.S.); m.m2132@yahoo.com (M.M.); dwli@njfu.edu.cn (D.L.); qzhuge@njfu.edu.cn (Q.Z.); 2 Clinical and Molecular Genetics Units, Institute of Child Health, London WC1N 1EH, UK; paulrutland51@btinternet.com

**Keywords:** secondary metabolites, biosynthetic pathways, *Populus*

## Abstract

*Populus* trees meet continuous difficulties from the environment through their life cycle. To warrant their durability and generation, *Populus* trees exhibit various types of defenses, including the production of secondary metabolites. Syntheses derived from the shikimate-phenylpropanoid pathway are a varied and plentiful class of secondary metabolites manufactured in *Populus*. Amongst other main classes of secondary metabolites in *Populus* are fatty acid and terpenoid-derivatives. Many of the secondary metabolites made by *Populus* trees have been functionally described. Any others have been associated with particular ecological or biological processes, such as resistance against pests and microbial pathogens or acclimatization to abiotic stresses. Still, the functions of many *Populus* secondary metabolites are incompletely understood. Furthermore, many secondary metabolites have therapeutic effects, leading to more studies of secondary metabolites and their biosynthesis. This paper reviews the biosynthetic pathways and therapeutic impacts of secondary metabolites in *Populus* using a genomics approach. Compared with bacteria, fewer known pathways produce secondary metabolites in *Populus* despite *P. trichocarpa* having had its genome sequenced.

## 1. Introduction

The genus *Populus* is a Northern Hemisphere willow family (Salicaceae), which includes thirty-five tree species. The North American poplar species can be categorized into three main groups: aspens, balsam poplars, and cottonwoods [1]. The soft poplar wood is used chiefly to make cardboard boxes, crates, paper, and veneers. Poplars proliferate but have relatively short lifespans. *Populus alba* and *Populus niger* are common Eurasian poplar species, whereas the balsam poplar (*P. balsamifera*) is native throughout North America [2].

The plants’ chemical syntheses are categorized as primary and secondary metabolites based on their chemical compositions and biosynthetic origin. A primary metabolite is directly implicated in average growth and usually has a physiological role in planta. On the other hand, secondary metabolic pathways produce many compounds that do not assist in plant growth but are necessary for the plant to thrive in its environment. Secondary metabolisms use the building blocks and biosynthetic enzymes originating from primary metabolism [3]. Secondary metabolites are classified based on their chemical structure and range of pharmacological activities.

**Figure 1:**
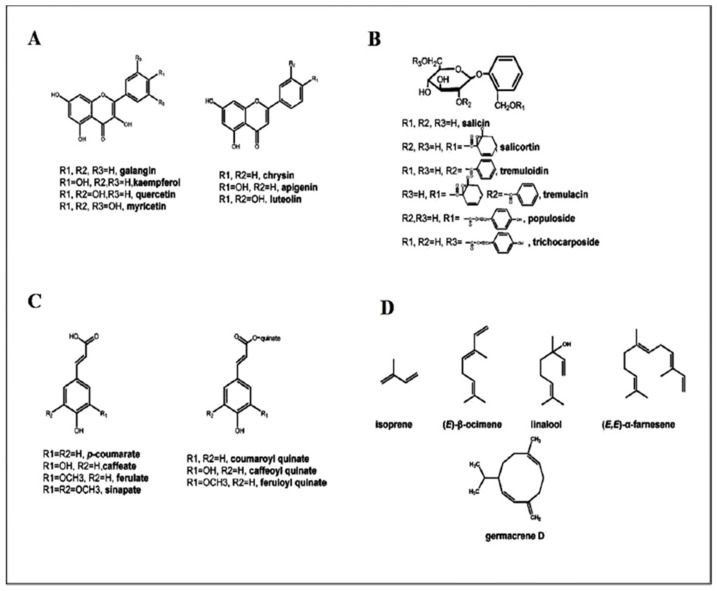
Structures of the critical types of secondary metabolites in *Populus*: (**A**) flavone and flavonol metabolites; (**B**) phenolic glycoside metabolites; (**C**) hydroxycinnamate, and (**D**) terpenoid metabolites.

Moreover, they can also be categorized based on their chemical composition and physiological function. The critical secondary metabolites, including phenolics, terpenoids, alkaloids, flavonoids, and glycosides, are actual examples of bioactive pharmacological compounds (Figure 1). Most secondary metabolites have a wide range of therapeutic activities interacting directly with cell receptors, membranes, and nucleic acids [3]. This review addresses the main secondary metabolite pathways with their cross-talk and the propriety of systematic metabolic modeling for understanding the secondary metabolites of *Populus*. Furthermore, it introduces new methods to understand *Populus* to explore its secondary metabolites and their medical application. The knowledge produced from this survey will increase our understanding of *Populus* secondary metabolites and provide a basis for their characterization and use.

## 2. Major Classes of Secondary Metabolites in Poplars

The present findings highlight that *Populus* bud ethanolic extract is a potent hepato-protective, anti-inflammatory, and NO-independent vasodilator of coronary arteries, indicating that it comprises metabolites with such actions. These findings compare favorably with other plants, such as *Fraxinus angustifolia*, *Pistacia lentiscus*, and *Clematis flammula*, whose antioxidant activities have been verified and whose total phenols, flavonoids, and tannins have been discovered in the related states [4]. Some of the major *Populus* secondary metabolites in this review have been presented in Table 1.

### 2.1. Shikimate–Phenylpropanoid Pathway

It has been shown that the principal secondary metabolites, such as lignans, hydroxycinnamates, anthocyanin, flavonoids, salicylic acid, tannins, and their esters originate from the shikimate–phenylpropanoid pathway [26,27]. It has also been shown that the biosynthesis of aromatic amino acids depends on the shikimate pathway in plants [28,29]. Therefore, its immediate end products can confirm the importance of the shikimate pathway behind primary metabolisms in plants. These include phenylalanine, which is a crucial feature in the phenylpropanoid pathway [28,29]. In the shikimate pathway, the first reaction commences with phosphoenolpyruvate and erythrose 4-phosphate to generate 3-deoxy-d-heptulosonate 7-phosphate.

On the other hand, shikimate production is directed by subsequent ring closure, dehydration, and reduction. Finally, shikimate is included in sequential reactions with shikimate kinase, chorismate synthase, arogenate dehydratase, and 5-enolpyruvylshikimate-3-phosphate synthase construct phenylalanine [30]. First, phenylalanine ammonia-lyase (PAL) plays an essential role in initiating the phenylpropanoid pathway. Regarding this, PAL catalyzes phenylalanine deamination into trans-cinnamic acid. Then, cinnamate 4-hydroxylase (C4H) catalyzes the hydroxylation of cinnamate into 4-coumarate. C4H is a cytochrome P450-dependent monooxygenase and is the first of three P450s involved in lignin biosynthesis. The next step produces the core compound of the phenylpropanoid pathway, 4-coumaroyl coenzyme A (CoA) (p-coumaroyl CoA). Then, 4-coumarate: CoA ligase (4CL) catalyzes the formation of the CoA thioester in an ATP-dependent reaction producing 4-coumaroyl CoA [28,30,31]. Subsequently, different enzymatic pathways produce essential metabolites, such as lignans, flavonoids, hydroxycinnamates, salicylic acid, tannins, and esters [26,27].

#### The Medical Importance of Phenylpropanoid Metabolites

To date, many phenylpropanoid metabolites exhibit medical benefits, including anti-retroviral [32], anti-hypertensive [33], anti-inflammatory [34], and insulin-sensitizing activities [35,36]. In addition, the oxidative effect reduces the risk of chronic diseases, such as cardiovascular disease [35], cancer [37], and osteoporosis, while inhibiting low-density lipoprotein oxidation [38] and having anti-aging effects [39].

- Flavonoids

Flavonoids are essential compounds produced in the phenylpropanoid pathway. Flavonoids have antioxidative, anti-inflammatory, anti-mutagenic, and anti-carcinogenic properties related to regulating necessary cellular enzyme functions [40,41]. Flavonoids inhibit enzymes such as aldose reductase, xanthine oxidase, phosphodiesterase, Ca2+ ATPase, lipoxygenase, and cyclooxygenase (COX) in neurodegenerative diseases, such as Parkinson’s [34]. Flavonoids also prevent the damages caused by free radicals, including the direct scavenging of free radicals. In addition, flavonoids are oxidized by radicals, resulting in more stable and less reactive radicals [42].

- Anthocyanidins and anthocyanins

Anthocyanins are reddish-colored water-soluble pigments endowed with a phenolic group. They are various in medical applications and beneficial health effects. In vitro and in vivo studies and human clinical trials have shown that anthocyanins and anthocyanidins are involved in antioxidative [43], antimicrobial [44], and neuroprotective activities [45]. Furthermore, various pathways are associated with the protective effects of anthocyanidins and anthocyanins, including the free-radical scavenging, COX, and mitogen-activated protein kinase pathway inflammatory cytokine signaling [46].

### 2.2. Phenolic Glycosides

The phenolic glycoside pathway is not fully characterized. However, several studies have shown that phenolic glycosides (PGs) are descended from cinnamic acid, involved in the shikimate–phenylpropanoid pathway, and made in the catalytic reaction phenylalanine and PAL [25]. Furthermore, PAL is inhibited by 2-aminoindan-2-phosphonic acid in the reaction above, reducing cinnamic acid production [47,48].

**Figure 2:**
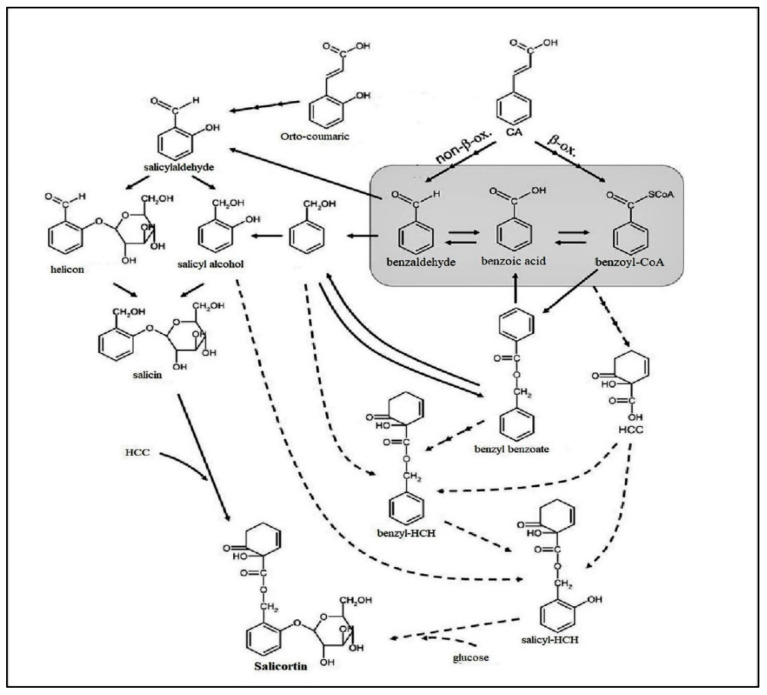
Salicortin biosynthesis pathway.

Phenolic glycosides can be generated by cinnamic acid in three methods. Cinnamic acid is catalyzed to produce o-coumaric acid to reform salicylaldehyde into salicyl alcohol and helicon in the first method. Thus, 1-hydroxy-6-oxo-2-cyclohexene-1-carboxylic acid and salicin react to form salicortin. In the second method, and according to Figure 2, cinnamic acid forms benzoyl-CoA through the β-oxidative (β-oxo) pathway. Further, benzoyl-CoA conjugates benzyl alcohol to hydrolyze benzyl benzoate creating mostly benzoic acid in the β-oxo pathway. In the third method, the benzoate pool may degenerate using two separate non-β-oxo pathways by turning cinnamic acid or phenylpyruvate directly into benzaldehyde. Both salicyl and hydroxy cyclohexenonoyl moieties may be descended from benzoate, resulting in benzoate pool regenerations as a regulator of salicortin biosynthesis (Figure 2) [48].

#### The Medical Importance of Phenolic Glycoside Metabolites

Salicortin is a bioactive component of *P. balsamifera* that actively represses adipogenesis in 3T3-L1 adipocytes, intimating possible anti-obesity activity [49]. Harbilas et al. [50] reported that salicortin diminishes obesity and features of metabolic syndrome and reduces retroperitoneal fat pad weights and hepatic triglyceride quantity while also improving the leptin, insulinemia, glycemia, and adiponectin contents. Harbilas et al. [50] also reported that the fundamental factors in the signaling pathways are connected with glucose control and lipid oxidation in the liver, muscle, and adipose tissue. Salicortin likewise beneficially inhibited adipogenesis in the 3T3-L1 cell line [49]. Furthermore, it has been shown that salicortin regulates body weight, retroperitoneal fat pad weight, insulin, liver lipid content, circulating glucose, and leptin levels [49]. Salicortin also initiates pathways incorporated with lipid oxidation, thermoregulation, and glucose metabolism [50].

A survey of the primary insulin-responsive tissues, adipose tissue, liver, and skeletal muscle, showed data highlighting implied mechanisms at multiple levels of metabolic direction. For example, salicortin significantly affects muscle p44/42 MAPK activation more than the expression of PPARα, leading to improved fatty acid oxidation and enhanced muscle insulin sensitivity [51]. Furthermore, alicorn duplicated liver Akt phosphorylation, an indispensable component of the insulin-signaling cascade [50]. An analysis of adipose tissue research revealed that salicortin diminished p44/42 ERK MAP kinase, showing a tendency toward weight reduction and the inhibition of clonal expansion of 3T3-L1 adipocytes. Moreover, the aim of developing adipose tissue Akt and CPT-1 expression supports the idea that salicortin enhances insulin-dependent lipid oxidative pathways [50].

### 2.3. Hydroxycinnamates

The lignin biosynthetic pathway produces hydroxycinnamates biosynthetically [52]. The reactions of O-methylation and hydroxylation, involving the cinnamate aromatic ring, form different hydroxycinnamates. The lignin biosynthesis converts the compounds (hydroxyl) cinnamyl alcohol dehydrogenase, (hydroxy) cinnamoyl-CoA reductase, 4-coumarate, and CoA ligase into their analogous hydroxycinnamoyl CoA, hydroxycinnamaldehydes, and hydroxycinnamoyl alcohols. It has also been shown that free hydroxycinnamic acids do not undergo hydroxylation and methylation. These outcomes suggest that hydroxycinnamates are top products isolated from the monolignol biosynthetic pathway [52,53]. It has been shown that 2–8% of the dry leaf weight in *Populus* was comprised of hydroxycinnamates and their derivatives [30]. In addition, different *Populus* species exhibited that 70% of the extra hydroxycinnamates and their derivatives play essential roles in the hydroxycinnamate pathway [30]. In *Populus*, phenylpropenoic acids include hydroxycinnamates, including ferulic acid, isoferulic acid, p-coumaric acid, caffeic acid, vanillic acid, p-hydroxybenzoic acid, esters, and aldehydes [30].

#### The Medical Importance of Hydroxycinnamates

In *Populus*, hydroxycinnamates and ferulic acid, including ferulic acid ethyl ester and curcumin, exhibit effective antioxidant activity by scavenging hydroxyl, superoxide, peroxyl radicals, peroxynitrite, and singlet oxygen, and reducing agents both in vitro and in vivo [54,55]. In addition, among the hydroxycinnamic acids, caffeic acid phenethyl ester and caffeic acid reveal the highest antioxidant activity, whereas p-coumaric acid had the least [56]. Hydroxycinnamic acids exhibit a wide range of possible therapeutic impacts in the therapy of cancer [57,58], diabetes [59,60,61], and kidney and cardiovascular diseases [62,63]. They also have hepato- [64,65], neuro- [66], photo-protective effects [67] and antimicrobial [68] and anti-inflammatory activities [69,70]. Vo et al. [71] investigated the anti-inflammatory mechanism of methyl p-hydroxycinnamate. They showed inhibiting enzymes suppressed pro-inflammatory mediators such as nitric oxide and prostaglandin E2 and iNOS and COX-2 protein expression. Moreover, methyl p-hydroxycinnamate significantly suppressed the lipopolysaccharide-induced degradation of IκB, which retains NF-κB in the cytoplasm, thereby inhibiting the pro-inflammatory transcription genes triggered by NF-κB in the nucleus. In addition to hydroxycinnamic acids, ferulic acid also exhibited anti-HIV effects and inhibited Gram-positive and -negative bacterial growth [72]. In addition, these compounds decreased the delivery and activity of the p24 antigen, an essential virus capsid protein, and inhibited the replication of the virus without cytotoxic effects [72].

### 2.4. Fatty Acids

Fatty acids are substrates for producing an extensive group of metabolites in plants, some of which are secondary metabolites. The best representatives of this assortment are a group of composites collectively termed green leaf volatiles, which involves C6 aldehydes, alcohols, and their esters [73]. Green leaf volatiles restrict the distinctive scent created when leaves are physically injured [74]. Five green leaf volatiles, (Z)-3-hexenol, (Z)-3-hexenylacetate, (Z)-3-hexenal, (E)-2-hexenal, and 1-pentenol, were described to be released from ozone-exposed *Populus* × *canescens* [75]. Frost et al. [76] reported that (Z)-3-hexenylacetate has been identified from insect-damaged *Populus* plants. The first level of green leaf volatiles production control happens at the step of lipid hydrolysis, which is usually triggered by tissue disruption. Lipid hydrolysis releases free fatty acids into the lipoxygenase (LOX) pathway [73]. Lipoxygenase catalyzes the dioxygenation of polyunsaturated fatty acids, such as linoleate and linolenate, to generate hydroperoxides in the lipoxygenase pathway [77]. Cheng et al. [78] showed that *P. deltoides PdLOX1* and *PdLOX2* are two lipoxygenase genes exhibiting predominant 13-LOX activity and up-regulated by fungal pathogens, mechanical damage, and exposure to methyl jasmonate that mimics insect feeding. Cheng et al. [78] also suggested that *PdLOX1* and *PdLOX2*, including in the green leaf volatiles development pathway, play a vital role in *Populus* resistance to environmental stresses. The hydroperoxides produced during LOX activity may assist as substrates for allene oxide synthase, leading to jasmonate formation, one crucial signaling molecule for plant protection [79]. Lawrence et al. [80] revealed that injury and insect herbivory in *Populus* induce the *hydroperoxide lyase* gene to fabricate volatile aldehydes and alcohols through the hydroperoxide lyase pathway metabolites.

#### The Medical Importance of Fatty Acids

Frequently, fatty acids are considered to have an immunomodulatory function. For example, they seem essential for the differentiation of T lymphocytes, indirect inflammasome activation, and the diminished excretion of some inflammatory cytokines [81]. In addition, short-chain fatty acids (SCFAs) are essential for intestinal homeostasis since they control proper microbiome dynamics by suppressing the generation of some bacteria at a low pH environment [82]. In addition, these acids influence immune reactions not only in the intestines but also in different tissues [83,84].

### 2.5. Terpenoid Pathway

**Figure 3:**
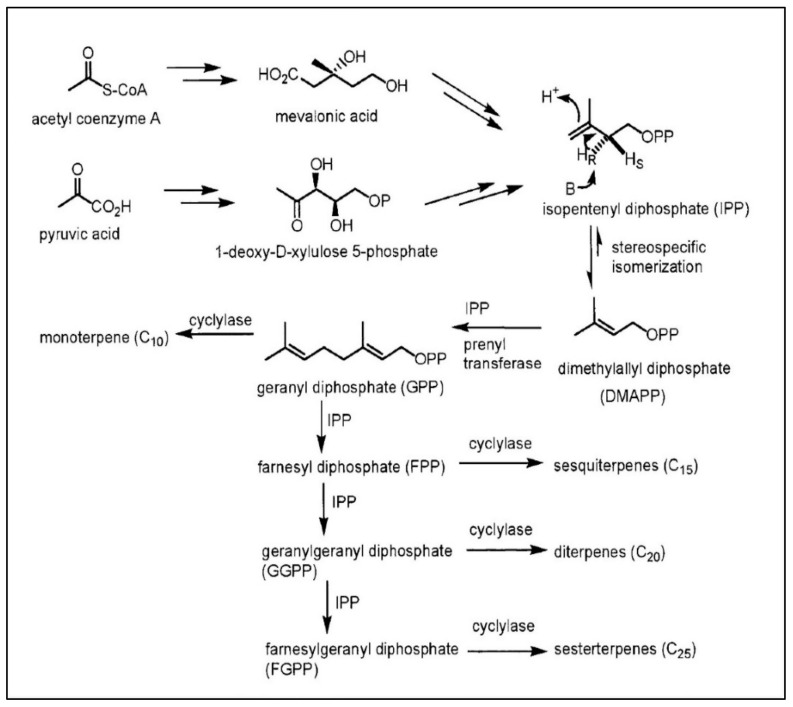
Terpenoid pathway.

The terpenoid pathway produces numerous secondary metabolites and is a crucial biochemical pathway in plants [85]. It affects the production of photosynthetic pigments, including chlorophyll and carotenoids, and plant hormones such as abscisic acid, gibberellins, brassinolides, and cytokinins [85]. There are two principal pathways in plants involved in terpene production: cytosol-localized mevalonate and plastid-localized methylerythritol phosphate (also called 1-deoxy-d-xylulose) [51]. Both pathways begin with the production of the C5 precursor isopentenyl pyrophosphate (IPP). First, IPP turns into the allylic isomer, termed dimethylallyl pyrophosphate (DMAPP). Next, IPP and DMAPP (the most specific and abundant product of the plant terpene family) regulate the development of geranyl pyrophosphate (GPP) and geranylgeranyl pyrophosphate (GGPP) via farnesyl pyrophosphate (FPP) and plastids in the cytosol (Figure 3). In this process, terpene synthases catalyze monoterpenes, diterpenes, and sesquiterpenes from GPP, GGPP, and FPP (Figure 3). All sesquiterpenes are constructed from FPP by the development of sesquiterpene synthase. Importantly, terpene synthase can provide multiple products from a single substrate [85].

#### The Medical Importance of the Terpenoid Pathway

The therapeutic effects of terpenes have been focused on their anti-cancer effects because the derivative Taxol is one of the most widely used anti-cancer compounds worldwide [85,86,87]. In addition to anti-cancer effects, these compounds have antimicrobial [88], antifungal [89], antiviral [90], anti-inflammatory [91] and antioxidant [92] attributes. Monoterpenes are the main components of essential oils and belong to the 10-carbon terpene group called isoprenes. The mechanism of the anti-cancer effect of monoterpenes is still not fully understood. Studies have suggested that monoterpenes suppress tumor growth by inhibiting the NF-κB pathway and inducing apoptosis [93]. The most striking effect of diterpenes is their anti-cancer effect, in addition to their effect on oxidative stress, inducing apoptosis and cell arrest, and inhibiting angiogenesis which prevents tumor growth. In addition, diterpenes exhibit cardiovascular and anti-inflammatory effects [94].

## 3. *Populus* Secondary Metabolites Dealing with Environmental Stresses

### 3.1. Microbial Pathogens

Microbial pathogens such as viruses (e.g., *Populus* mosaic virus), bacteria (e.g., *Xanthomonas popular*), and fungi (e.g., *Melampsora* species) cause a variety of infections in *Populus* [95]. The accumulation of secondary metabolites results in toxicity to microbial pathogens and can act as a biological defense. pyrocatechol, benzoic acid, and salicylic acid isolated from the *P. tremuloides* leaves and bark were determined to repress the increase of *Hypoxylon pruinatum* [96]. These pyrocatechol syntheses had a potent inhibitory influence on the growth of various fungal pathogens and performed as phytoanticipins because they are constitutively present in the owner tissues and effective before the fungal invasion [97]. In addition, *P. tremuloides* has been shown to synthesize secondary metabolites, including phenolic glycosides, which inhibit spore germination of two fungi, *H. mammatum* and *Alternaria* spp. [97]. Additionally, tannins are also shown to be included in *Populus* defense against microorganisms. It has been shown that tannins perform as resistant chemicals to bacterial and fungal pathogens [98]. The phytochemical analysis verified that delaying tannin synthesis increased infected leaves in *P. trichocarpa* × *deltoides* [99]. In addition, the lignin pathway may confer a similar defense role. For example, some intermediates of lignin, such as monolignols, may directly influence infecting pathogens [100].

### 3.2. Herbivores

*Populus* trees are always facing different kinds of insect herbivores, including sucking pests (meadow spittlebug), boring pests (*Saperda calcarata*), and leaf feeders (forest tent caterpillar). These bug herbivores could produce a widespread decline in the growth and productivity of *Populus*. Therefore, *Populus* producing and accumulating a myriad of secondary metabolites acts as a defense against pest herbivory. Two phenolic glycosides, salicortin and tremulacin, have been proven to be potential herbivore toxins. In addition, they have been confirmed to have substantial adverse effects on herbivore growth and development [101]. High densities of phenolic glycosides increase herbivore development time and reduce herbivore weight [102]. It has been shown that the aspen-isolated *dihydroflavonol reductase* (DFR) gene, which is involved in condensed tannin synthesis and concentrations, was significantly induced by pest treatment. Therefore, it is recommended that in *Populus*, the condensed tannins may be necessary for defending against herbivores [103]. Behnke et al. [75] proved that herbivory damage causes the release of some *Populus* metabolites, including monoterpenes, sesquiterpenes, homoterpenes, and green leaf volatiles. Arimura et al. [104] suggested that in *Populus*, insect-induced volatiles metabolites appear as a natural defense because of the toxicity against the attacking pests. In addition, it has been shown that *P. balsamifera* utilizes multiple secondary metabolites in various parts as antifeedants in protection from snowshoe hare (*Lepus americanus*) [105]. Reichardt et al. [105] also showed that cineol, benzyl alcohol, and (+)-bisabolol have been associated with defense in buds. Moreover, 6-Hydroxycyclohexenone and salicaldehyde have been associated with the defense in internodes. It was recognized that the concentration of 6-Hydroxycyclohexenone could be achieved by the hydrolysis of phenol glycosides when *Populus* tissues are damaged, suggesting a *Populus* dynamic chemical defense toward environmental stresses.

### 3.3. UV-B Protection

Solar ultraviolet-B (UV-B) radiation can diminish and induce mutations in macromolecules, nucleic acids, and proteins [106]. Yang et al. [107] reported that UV-B radiation mainly affects physiological changes in plants, including leaf area, reduction in photosynthetic rate, damage to photosystem II, biomass accumulation, and reduction in height. Several studies proved that the protection from UV-B radiation results from the increase of phenolics, as UV-absorbing composites diminish the damage by extreme UV-B radiation in *Populus* [108]. In *Populus*, secondary metabolites hydroxycinnamates, glycosylated flavonols, and chlorogenic acid are effective UV-absorbing composites [109]. These composites assembled at the epidermal layer, suggesting they may operate as epidermal attenuation of UV-B in *Populus* [109].

### 3.4. Drought and Temperature Stresses

Metabolic changes play vital roles in plant acclimatization and adjustment to temperature stresses. The secondary metabolite isoprene has also been recommended to preserve leaf physiological processes during high-temperature stress. Behnke et al. [110] showed that *Populus* clones that do not release isoprene exhibited diminished absorption and photosynthetic electron transportation rates throughout heat stress, confirming that isoprene emission is associated with thermotolerance photosynthesis. Plants, particularly woody perennials, need to deal with seasonal irregularities in water availability. Some plants tolerate lack of water by storing or expanding high concentrations of primary metabolites, including organic acids, amino acids, and sugars [111]. Secondary metabolites have also been involved in plant protection upon drought stress. Hale et al. [112] observed an increased concentration of phenolic glycosides in response to drought stress in *Populus*.

## 4. Conclusions

*Populus* trees generate many secondary metabolites that are possibly implicated in interfering with a complex mixture of optional and necessary interactions with biotic factors from symbioses to pathogenicity [113]. In addition, several of these secondary metabolites may be included in the *Populus* protection to abiotic stresses. Knowledge of secondary metabolite biosynthesis and its biological functions will present novel information and tools for trait genetic enhancement. Therefore, it is required that *Populus* will have an improved utilization as a bioenergy feedstock. Unfortunately, many *Populus* cultivars are sensitive to pathogens [95]. Because *Populus* plantations are complicated ecosystems contributing to virtual environments for various organisms, widespread utilization of pesticides may not be available [114]. Hence, it is necessary to develop resistant *Populus* trees.

Furthermore, if *Populus* trees are widely utilized as a bioenergy feedstock, they must develop in diverse environments where the trees may encounter severe weather situations. Therefore, tolerance to temperature and drought stresses will be critical. Again, secondary metabolism performs a fundamental target for genetic improvement of *Populus* defense to such abiotic stresses. In using *Populus* as a feedstock, one crucial restriction is cell wall recalcitrance, of which lignin is the principal factor. Thus, overcoming lignin content has been a practical approach to improve saccharification yields [115]. It is, therefore, necessary to fully understand the biosynthesis of lignin and its control. Equally relevant, we need to recognize how the adjustment of lignin content and structure will affect *Populus*–environment interactions and their effect on *Populus* productivity. Differences in secondary metabolite profiles among various genotypes of *Populus* have been recognized [116]. It will also be exciting to discover associations between particular chemical profiles with particular environmental factors correlated with particular ecosystems, producing novel insights into the development of secondary metabolism. In addition, a thorough knowledge of biosynthesis and the role of secondary metabolites in *Populus* [117] will produce excellent references for the understanding of secondary metabolism in other tree species, which may direct to stimulate the genetic improvement of trees.

## Figures and Tables

**Table 1:** Major classes of secondary metabolites in poplars and *Populus* species applied in these studies.

Secondary Metabolite Product	*Populus* Species	Reference
Shikimate–Phenylpropanoid	*Populus trichocarpa* *Populus tremula × Populus alba*	[5,6]
Flavonoids	*Populus tomentosa* *Populus deltoides* *Populus trichocarpa*	[7,8,9,10]
Anthocyanins	*Populus deltoides* (*sp. Linn*) *Populus deltoids*	[11,12]
Phenolic Glycosides	*Populus davidiana*	[13]
Hydroxycinnamates	*Populus tomentosa* *Populus tremuloides*	[14,15]
Fatty acids	*Populus trichocarpa* *Populus nigra* *Populus × euramericana* *Populus deltoides*	[16,17,18,19]
Terpenoid	*Populus euphratica* *Populus × canescens* *Populus trichocarpa* *Populus × euramericana* (*sp. Nanlin*)	[20,21,22,23,24,25]

## Data Availability

Not applicable.
